# Routine data-based quality indicators for the treatment of gonarthrosis and coxarthrosis patients in the ambulatory care sector – A study protocol for a cluster-randomised pilot trial to evaluate the MobilE-ARTH study

**DOI:** 10.1186/s12891-022-05699-7

**Published:** 2022-08-04

**Authors:** Tobias Bock, Ronja Flemming, Wiebke Schüttig, Anja Schramm, Martin B. Weigl, Leonie Sundmacher

**Affiliations:** 1 Chair of Health Economics, Department of Sport and Health Sciences, Technical University of Munich (TUM), Munich, Germany; 2 Department of Analytics and Data, AOK Bavaria – The health insurance fund, Munich, Germany; 3 Department of Orthopaedics and Trauma Surgery, Musculoskeletal University Centre Munich (MUM), University Hospital, Ludwig Maximilian University of Munich (LMU), Munich, Germany

**Keywords:** Gonarthrosis, Coxarthrosis, Quality indicators, Quality of care, Ambulatory care, Networks, Feedback, Coordination of care, Continuity of care, Cluster-randomised trial

## Abstract

**Background:**

In 2019, Germany had the highest rate of hip replacement surgery and the fourth highest rate of knee replacement surgery among more than 30 OECD countries. The age-standardised rates were estimated at 174 hip joint and 137 knee joint replacements per 100,000 population. Against this background, the contrast between financial incentives for surgery and missing incentives for non-surgical treatment options is repeatedly discussed. Quality indicators (QIs) can serve to measure and transparently present the quality of evidence-based care. Comparing results in the form of audit and feedback has been shown to improve e.g. guideline-compliant ambulatory care. Existing QIs targeting the care of gon- and coxarthrosis mainly focus on discharge management after joint replacement surgery and/or require additional data collection. Therefore, as part of the MobilE-ARTH project, a set of QIs for ambulatory care prior to joint replacement surgery calculable based on routine data is being developed. The present study’s aim is to evaluate the impact of this QI set in terms of providing feedback on the quality of care.

**Methods:**

The MobilE-ARTH project comprises (Phase 1) developing a QI set following the RAND/UCLA Appropriateness Method, (Phase 2) implementing the QIs in established physician networks of a German statutory health insurance (SHI) within a prospective, non-blinded, cluster-randomised pilot study, and (Phase 3) evaluating the QI set’s effectiveness. The physicians in the intervention networks will (a) receive feedback reports providing information about the routine data-based QIs of their gon- and/or coxarthrosis patients and aggregated results for their network, and (b) be invited to two voluntary, facilitated network meetings. In these network meetings, the physicians can use the information provided on the feedback reports to discuss multiprofessional care pathways for patients with gon- and/or coxarthrosis. Selected indicators of the QI set will serve as primary and secondary outcome measures. Routine data will be analysed within multi-level models using an intention-to-treat approach.

**Discussion:**

Feedback reports help maintaining clinical standards and closing the gap between evidence and medical practice, thus enabling an overall improvement in health care. Providing physicians with QI-based information on quality of care promotes identifying strengths and weaknesses in medical treatments.

**Trial registration:**

German Clinical Trials Register, number DRKS00027516, Registered 25^th^ January 2022 – Prospectively registered.

## Background

Osteoarthritis is one of the most frequent joint diseases worldwide and it has a significant impact on both patients' quality of life and health care systems [1–4]. It is estimated that in 2019, gon- and coxarthrosis resulted in 18.9 million years of life lived with physical impairment (Years Lived with Disability (YLD)) globally [5]. In 2015, 48.1% of women and 31.2% of men over the age of 65 were affected by osteoarthritis in Germany, and the prevalence is expected to rise due to the ageing population [6].

Treatment options for gon- and coxarthrosis can be divided into conservative therapies (pharmacological and non-pharmacological treatments) and surgical treatments [7–11]. Surgical options include arthroplasty, osteotomy, resecting arthroscopic procedures and repair of the damaged articular cartilage [9, 11]. Endoprosthetic replacement of the affected joint is an effective measure to improve functionality and relieve pain [8]. However, in order to reduce the burden on patients and the health care system, joint replacement should only be considered as a last option after other conservative therapies have failed [12, 13].

Among more than 30 OECD countries, Germany was the country with most hip replacement surgeries and among the top four in terms of knee replacement surgeries in 2019 [14]. Germany achieved an age-standardised rate of 174 hip joint replacements and 137 knee joint replacements per 100,000 population [15]. In addition to the individual disease burden of the patients, osteoarthritis-related diseases are associated with a high and partly inappropriate utilisation of health care and consequently lead to considerable direct and indirect costs (e.g. incapacity for work, invalidity, early retirement) for the health care system as well as the economy [4].

Hence, an ongoing debate has emerged concerning whether these high joint replacement rates are the result of over-utilisation or whether they simply reflect the needs of an ageing population [13–16]. In this context, evidence of significant geographical disparities [16–18] and misguided economic incentives for surgical treatment are repeatedly discussed [17–19]. One possible explanation for an inappropriate utilisation of health care in the context of gon- and coxarthrosis might be the organisation of conservative treatment, which is mainly provided in the ambulatory care sector. The ambulatory care sector in Germany is characterised by a high density of office-based physicians and there is no system of gatekeeping that regulates access to and coordinates treatment by specialised physicians [20]. Patients are free to seek multiple opinions on a diagnosis or treatment without the need for referral. In addition to the freedom of choice, coordination of care is further complicated by the involvement of multiple physicians and their disciplines in the treatment of one patient. Overall, there is an urgent need for a well-organised and coordinated provision of effective and efficient treatment for patients with gon- and/or coxarthrosis.

Quality assurance is essential to offer an appropriate as well as cost-effective care to patients in line with the current state of medical progress. In this context, quality is represented based on the three dimensions of structural, process and outcome quality. The structural quality includes infrastructural, organisational and personnel conditions of the medical institution. Process quality encompasses all procedures of the care pathways along which medical services are provided. Within the framework of outcome quality, the result of the medical treatment is recorded. Since these dimensions are directly interrelated, simultaneous consideration is needed in terms of comprehensive quality assurance. Quality indicators (QIs) with specific norms and standards for each relevant aspect of medical care are used to measure and transparently present the quality of care and identify potential for improvement [21].

Especially in the case of chronic diseases such as osteoarthritis, the need for better coordinated ambulatory care has been widely acknowledged. In Germany, requirements for Disease Management Programs (DMPs) have already been implemented for other chronic diseases (e.g. diabetes mellitus or coronary heart diseases) to improve the quality and continuity of care for patients [22]. Even though DMPs for osteoporosis and rheumatoid arthritis have been approved and are currently being developed, there exists no structured care plan for osteoarthritis patients.

Beside these disease-specific care plans, measures to improve the cooperation and exchange of information between ambulatory physicians have been established, such as the QuATRo (Quality in Physician Networks – Transparency with Routine Data) physician networks by one of the largest German statutory health insurance (SHI) organisations, Allgemeine Ortskrankenkasse (AOK). QuATRo is a project that enables regional and nationwide comparisons of the quality of health care between the participating networks as well as implementing standard care plans. Part of the project is to calculate eligible routine data-based indicators of the Quality Indicator System for Ambulatory Care (QISA). This indicator set has been compiled by the Institute for Applied Quality Improvement and Research in Health Care GmbH (aQua Institute) as a scientific cooperation partner of the Federal Association of the AOK [23].

Within the framework of the QuATRo project, participating ambulatory physicians are provided with data on quality indicators (QIs) in the form of feedback reports to enable a transparent view on the quality of care within their networks [24, 25]. The feedback reports aim to maintain clinical standards and close the gap between evidence and medical practice, so that an overall improvement in health care can be achieved [26–28]. Based on the proven positive effect of audit and feedback in the medical context [29, 30], it is assumed that the provided information on the quality of care promotes identifying strengths and weaknesses in medical care. Additionally, feedback is most effective when it is provided more than once and when communication takes place both in writing and through a peer review process [29, 30]. Therefore, the second important component of the QuATRo project is the organisation of regular meetings within the networks, which follow the principle of quality circles. In order to further improve the coordination and quality of care and encourage guideline-oriented care, the network physicians evaluate the results of the QIs included in the feedback reports and discuss possible options for action within their meetings [24].

Although quality monitoring is of high current relevance for effective and efficient ambulatory care [31], there are so far only a few QIs for the treatment of gon- and/or coxarthrosis. Apart from the indicator sets of the aQua Institute, which focus on the discharge management of patients after joint replacement surgery, and a small set of the National Association of SHI Physicians (Kassenärztliche Bundesvereinigung (KBV)) with four indicators that refer to osteoarthritis in general and mostly require the collection of clinical data [32], there is no set of QIs for measuring the ambulatory care of patients with gon- and/or coxarthrosis prior to joint replacement surgery based on routinely collected claims data.

The MobilE-ARTH project aims to close this significant gap to contribute to a quality- and thus patient-oriented ambulatory care of gon- and coxarthrosis. It includes (Phase 1) systematically developing a QI set that can be calculated based on routinely collected data sets of SHIs, following the structured RAND/UCLA Appropriateness Method [33], (Phase 2) implementing this QI set in the form of feedback reports and facilitated network meetings in the established QuATRo networks within a cluster-randomised study, and (Phase 3) evaluating the effectiveness of providing ambulatory physicians with this validated set of QIs for the treatment of patients with gon- and/or coxarthrosis.

The hypothesis of the study is that providing ambulatory physicians with information in the form of QI feedback reports on the gon- and coxarthrosis care, combined with the opportunity to discuss the given information with colleagues in facilitated network meetings, contributes to improving ambulatory care for patients with gon- and/or coxarthrosis.

## Methods

**Fig. 1:**
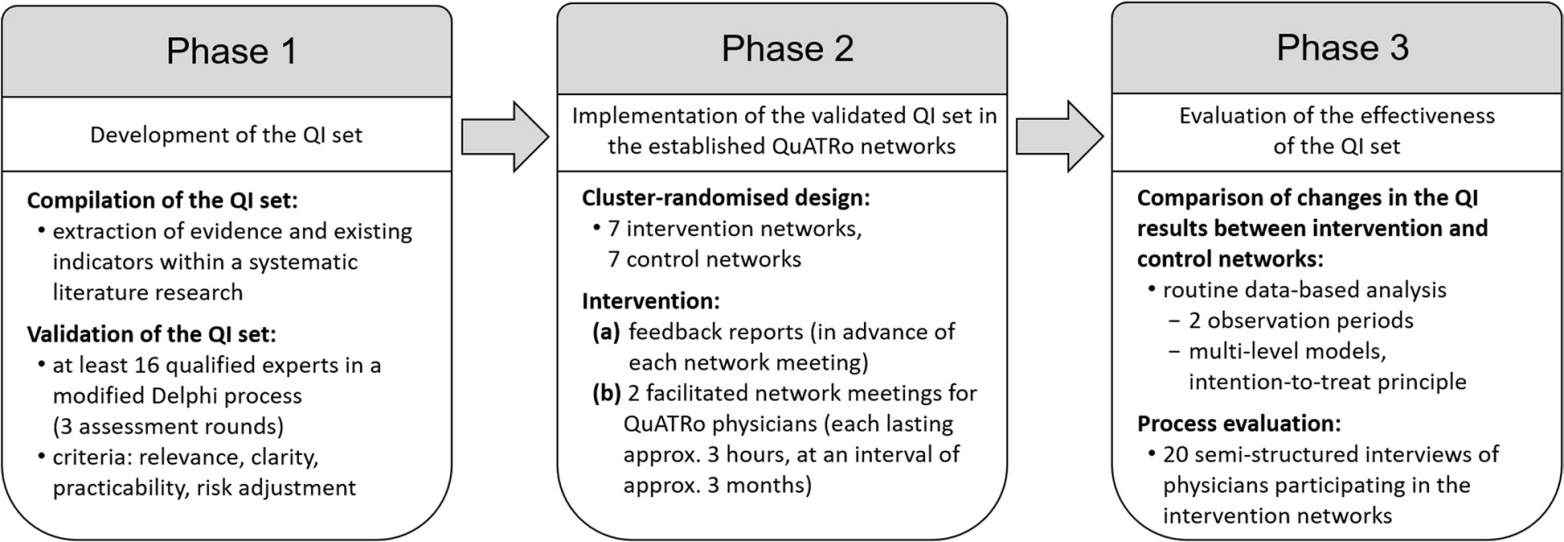
Flow chart of the design of the MobilE-ARTH project

The MobilE-ARTH project is to be planned and carried out by a study team of the Chair of Health Economics of the Technical University of Munich (TUM). This protocol describes a three-phase project design (see Fig. 1) using the model of an existing protocol for developing QIs [34].

### Phase 1: Development of the QI set

(This phase started in 2021 and is ongoing)

#### Aim

The aim of the first project phase is to develop a QI set calculable on routine data for the ambulatory care of patients with gon- and/or coxarthrosis prior to the implantation of an endoprosthesis.

#### Design

Adhering to the RAND/UCLA Appropriateness Method, the approach for developing the QI set comprises a systematic literature review with a subsequent validation by an expert panel in a modified Delphi process.

The systematic literature research is performed to identify, synthesise and critically evaluate evidence on treatment options and existing QIs for the ambulatory care of patients with gon- and/or coxarthrosis. A detailed search strategy is applied to five key electronic databases (Pubmed, Cochrane Library, Web of Science, Scopus and PEDro) for English or German publications from January 2000 to June 2021. Two reviewers independently select studies for inclusion, and disagreements are resolved through consensus. Guidelines, systematic reviews and randomised trials that evaluate the effect of conservative therapies (pharmacological and non-pharmacological treatments) for gon- or coxarthrosis are included. Publications evaluating surgical and post-surgical interventions as well as publications evaluating therapy options that are not calculable on routine data are excluded. As the result of the literature review, a set of fully operationalised QIs will be developed. According to the methodological recommendations of the aQua Institute [35], a specification sheet will be prepared for each QI with information on the underlying evidence as well as the details of the indicators’ calculation.

In order to validate the developed QI set, a modified Delphi process with at least 16 qualified representatives from different medical disciplines relevant for the ambulatory treatment of gon- and coxarthrosis (general medicine, orthopaedics, orthopaedic surgery, radiology, rehabilitation medicine, alternative medicine, physiotherapy) will be carried out. The process will comprise three assessment rounds, the first two of which will take place via e-mail, while the third will be a panel meeting. The panel meeting will take place virtually for one day under the leadership of a facilitator experienced in using the RAND/UCLA Appropriateness Method. For the three assessment rounds, background information, the specification sheets for the QIs and quantified examples will be presented to the members of the panel. The experts will rate each QI on a scale of 1 to 9, where 1 means that the QI is very inappropriate for the quality assessment of gon- and/or coxarthrosis care, and 9 means that the QI is very appropriate for this purpose. With respect to the following criteria, the expert panel will discuss the available evidence and select the most appropriate QIs with suggestions for modification if required:

- Relevance: The QI contains information that holds importance for patient care and the health care system.
- Clarity: The QI is able to holistically and comprehensively measure the quality aspect that it aims to measure.
- Practicability: The QI requires only routine data to be calculated and is clearly interpretable for the physicians.
- Risk adjustment: The QI considers the different factors of the patient structure of ambulatory practices to enable a fair comparison of quality of care.

The result of this first phase will be a set of QIs monitoring the ambulatory care of patients with gon- and/or coxarthrosis, which can be calculated based on routine data.

### Phase 2: Implementation of the validated QI set in the established QuATRo networks

#### Aim

In the second part of the project, the consented QI set will be implemented in the established QuATRo physician networks of the AOK Bavaria by providing the participating physicians with feedback reports and inviting them to facilitated network meetings.

#### Design

The field-testing phase of the MobilE-ARTH project is a prospective, non-blinded, multicentre, cluster-randomised pilot study (with unequal cluster size).

##### Study setting

The QuATRo physician scheme in Bavaria comprises about 700 physicians in 14 networks that will be randomised to seven intervention networks (which will receive feedback reports and be invited to facilitated network meetings) and seven control networks (usual care) to enable subsequent evaluation of the effect of the intervention on patient care.

##### Randomisation

In order to evaluate the effectiveness of the QI set, the 14 established QuATRo physician networks are randomly allocated to either the intervention or control group in a 1:1 ratio. Every network comprises about 50 physicians on average, leading to about 350 physicians in the seven intervention networks to be invited to participate in the intervention. In order to reach a sufficient number of participating physicians in every intervention network, the invitation will provide a detailed explanation on the aims of the study as well as the entire project in order to highlight the possible benefits for gon- and/or coxarthrosis care. The randomisation will be conducted by the study team and will not be blinded due to the design of the intervention.

##### Participants

Physicians are included in the study process if they fulfil the following criterion:

- Affiliation to one of the intervention networks of the QuATRo physician scheme of the AOK Bavaria (a physician cannot belong to two or more networks)

Patients fulfilling the following characteristics are included in the study and will be analysed for the feedback reports and evaluation:

- 18 years or older
- Diagnosis of gonarthrosis (ICD-10-GM M17) and/or coxarthrosis (ICD-10-GM M16) at least once during the respective observation period
- Continuous insurance with the AOK Bavaria (at least 350 days within the respective observation period)
- Continuous registration in one of the 14 QuATRo physician networks of the AOK Bavaria (at least 350 days within the respective observation period)

Current data of the AOK Bavaria indicate that around 1,000 patients on average can be expected to meet these characteristics in each of the networks in both observation periods. Patients are excluded in case of death during the respective observation period.

##### Intervention

The implementation of the QI set into the intervention networks will be realised by (a) providing the participating ambulatory physicians with feedback reports and (b) organising facilitated network meetings to enable exchange among the ambulatory physicians.

- Feedback reports
  At the beginning of the intervention phase, the QI set will be calculated by the study team based on the network-specific routine data provided by the AOK Bavaria for the patients with gon- and/or coxarthrosis of the participating physicians from the respective intervention networks. In the next step, the study team will prepare the results of the QI calculations individually for each physician’s practice and for each intervention network in the form of a feedback report. These feedback reports will be sent to the management of every intervention network via the AOK Bavaria, which will provide these for the corresponding physicians.
- Network meetings
  Subsequently to the provision of the QIs in the feedback reports, the physicians will be invited to two voluntary, facilitated network meetings, each lasting approximately three hours, at an interval of approximately three months. These meetings will be organised individually for each intervention network. The management of the respective intervention network will be contacted by the study team via the AOK Bavaria. The physicians will then be invited to these meetings by their respective QuATRo network management. The network meetings will be led by a facilitator who is experienced and trained in the contents and will give the physicians the opportunity to exchange information with colleagues from the respective QuATRo network about the care situation of patients with gon- and/or coxarthrosis. The feedback reports will be used by the physicians in the network meetings as a basis for identifying the well-functioning aspects as well as discussing solution approaches for possibly existing factors in need of improvement concerning quality of care to jointly develop care pathways for gon- and/or coxarthrosis patients within the QuATRo networks.

### Phase 3: Evaluation of the effectiveness of the QI set

#### Aim

In order to evaluate the effectiveness of the QI set, the quality of care in the intervention and control networks will be compared using the QI results. In addition, a process evaluation will be conducted to assess the feasibility of the QIs for routine care and identify possible facilitators or barriers for the implementations of the intervention.

#### Design

##### Data collection

The data used for the quantification of the QIs are secondary data, in particular routinely collected claims data provided by the AOK Bavaria. In order to evaluate the effect of the intervention, routine data from two observation periods – each lasting one year – will be used for the MobilE-ARTH project. Due to the impact of the Covid-19 pandemic, the first observation period is from January until December 2019 to ensure a view on gon- and coxarthrosis care under non-pandemic conditions concerning e.g. the fact that people were only allowed to make an appointment with a physician in acute situations or medical emergencies during the lockdown phase [36]. The second observation period will comprise the year after the implementation of the QI set in the intervention networks.

##### Outcomes

The QI set will serve to measure the effectiveness of the intervention for the ambulatory care of gon- and coxarthrosis. The validation of the QI set will therefore include the definition of indicators, which should be used as primary or secondary outcomes for the evaluation of the intervention study. Per definition of the study, all of the QIs will be calculable based on routinely collected claims data of SHI.

##### Data analysis

After validating the routine data, the QIs will be calculated for the two observation periods (before and after the intervention) in the intervention and control networks to evaluate the effect of the intervention.

Descriptive statistics will be used to outline the characteristics of the networks and the corresponding patient populations. The differences between the intervention and control networks according to the primary and secondary outcome measures will be evaluated within multi-level models using the intention-to-treat principle.

Because osteoarthritis is more frequent in women [4], gender correlates with morbidity risk [37] and women and men use different coping strategies for chronic health conditions [38], gender is adjusted alongside age by default – where indicated – in quantitative analyses. These gender differences will also be considered within the interpretations of the findings.

Additionally, a sensitivity analysis of the evaluation outcomes that encompass systematic comparisons of competing risk adjustment models and different significance levels will be conducted.

##### Process evaluation

In addition to the evaluation of effectiveness, a process evaluation will be conducted to assess the feasibility of the QIs in standard care. For this purpose, 20 semi-structured interviews of physicians participating in the intervention networks are planned. In order to investigate the clinical suitability, attitudes towards and satisfaction with the design of the QIs will be assessed.

## Discussion

Although continuous quality monitoring is crucial for effective and efficient ambulatory care [31], no QI set for measuring the ambulatory care of gon- and coxarthrosis prior to joint replacement surgery on the basis of routinely collected claims data has yet been developed. Through (a) the feedback of the routine data-based QIs and (b) the productive exchange at the network meetings, the MobilE-ARTH study will systematically reveal the reality of the ambulatory care of gon- and coxarthrosis to the participating physicians.

Given that a single QI is unable to cover all dimensions of quality of care there is a need for a balanced set of QIs to enable a transparent view on the entire care pathways for patients. It is expected that differences between the intervention and control networks will be evident with respect to measures of coordination and process quality. Due to the complex and long progression of disease [39], only small changes in outcome indicators can be expected.

The advantage that the QIs can be evaluated based on routine data without the need for additional data collection will ensure the practicability and efficient future use of the QI set [40]. In this regard, it is also important to consider that routine data do not usually contain clinical information, so it is possible to receive useful information on the coordination of care, but the QIs cannot enable a profound assessment of disease progression.

Given that the intervention will only take place in certain regions in Bavaria, the results cannot be transferred to other regions without restrictions. Another limitation is the short follow-up of the intervention considering the slow progression of the disease. It is assumed that long-term effects of the intervention can only be measured within a longer observation period. Initial effects concerning e.g. the prescribing behaviour are expected to be measurable within the follow-up period.

The MobilE-ARTH project will contribute to the systematic development of a QI set for audit and feedback in routine care. The QI set will specifically offer a supporting instrument to implement multiprofessional care pathways to improve the quality of the ambulatory care and coordination of patients with gon- and/or coxarthrosis and shift care to less cost-intensive service areas.

## Data Availability

The data that support the findings of this study are available from the AOK Bavaria but restrictions apply to the availability of these data, which were used under license for the current study, and so are not publicly available. Data is however available from the authors upon reasonable request and with permission of the AOK Bavaria and their regulatory authority.

## Abbreviations

AOK: Allgemeine Ortskrankenkasse (statutory health insurance in Germany)
aQua Institute: Institut für angewandte Qualitätsförderung und Forschung im Gesundheitswesen GmbH (Institute for Applied Quality Improvement and Research in Health Care GmbH)
DMP: Disease Management Program
IQTIG: Institut für Qualitätssicherung und Transparenz im Gesundheitswesen (Institute for Quality Assurance and Transparency in Health Care)
KBV: Kassenärztliche Bundesvereinigung (National Association of Statutory Health Insurance Physicians)
QI: Quality Indicator
QISA: Quality Indicator System for Ambulatory Care
QuATRo: Qualität in Arztnetzen—Transparenz mit Routinedaten (Quality in Physician Networks—Transparency with Routine Data)
SHI: Statutory Health Insurance
TUM: Technical University of Munich
YLD: Years Lived with Disability
